# Association of Histopathologic Characteristics in Diagnostic Prostate Biopsies With Decipher Genomic Classifier Risk Groups

**DOI:** 10.7759/cureus.113243

**Published:** 2026-07-23

**Authors:** Selin Kurt, Ayesha Usman, Emily Torres, Garrison Pease

**Affiliations:** 1 Pathology, Albert Einstein College of Medicine, Montefiore Medical Center, New York City, USA

**Keywords:** decipher genomic classifier, prostate adenocarcinoma, prostate biopsy, prostate cancer, risk stratification

## Abstract

Prostate cancer remains the most frequently diagnosed malignancy in men. While traditional clinicopathologic parameters guide management, genomic classifiers such as the Decipher assay are increasingly used to refine risk stratification.

In this study, we evaluated the association between Decipher genomic classifier (GC) (GenomeDx Biosciences, Vancouver, BC, Canada) risk groups and histopathologic features in diagnostic prostate biopsy specimens within a routine clinical setting.

We retrospectively reviewed 73 prostatic adenocarcinoma biopsies that underwent Decipher genomic testing, sampled between 2024 and 2025. Decipher risk groups demonstrated a significant association with grade groups (GG) (p=0.03 (χ²=16.6)), with high-risk (HR) cases showing a higher prevalence of higher GG compared to low-risk (LR) cases. Quantitative metrics of tumor burden were also significantly associated with risk groups: the mean positive core ratio (LR: 0.44 versus HR: 0.64; p=0.003) and mean maximum tumor length ratio (LR: 0.47 versus HR: 0.67; p=0.01) were significantly higher in the HR group. Aggressive features such as extraprostatic extension and intraductal carcinoma were identified exclusively in the HR group. No significant differences were observed for prostate-specific antigen (PSA) levels across groups.

In conclusion, histopathologic indicators of tumor aggressiveness and burden in diagnostic prostate biopsies show significant association with Decipher GC risk groups, supporting their complementary role in risk stratification.

## Introduction

Prostate cancer is the most prevalent malignancy among men, accounting for nearly one-third of all newly diagnosed cancers, and the second leading cause of cancer-related death after lung cancer [1,2]. Traditionally, clinical management and prognostic decision-making in prostate cancer have been based on key clinicopathologic parameters, including patient age, overall health status, serum prostate-specific antigen (PSA) levels, pathologic staging (TNM classification system), histologic grading (Gleason scoring system), and single biomarkers such as Ki-67 [3-5].

In recent years, the management of prostate cancer, similar to other malignancies, has evolved with the integration of molecular and genomic profiling tools, which enhance risk stratification and enable more personalized therapeutic approaches. Genomic profiling tools utilizing scoring systems have been classified into two broad categories: polygenic risk scores, which predict susceptibility via germline variations, and tumor-derived genomic classifiers (GCs), which evaluate gene expression patterns within tumor specimens [6].

The Decipher genomic classifier (GenomeDx Biosciences, Vancouver, BC, Canada) is a 22-gene assay utilizing microarray analysis of whole-transcriptome RNA expression from formalin-fixed, paraffin-embedded (FFPE) prostate tumor tissue. By stratifying patients into low-, intermediate-, and high-risk categories, Decipher GC was initially validated to predict metastatic risk following radical prostatectomy. Subsequent research has demonstrated its superior prognostic accuracy relative to traditional clinicopathologic variables, establishing it as one of the most broadly adopted genomic tests in clinical practice. Accordingly, it has been incorporated into the National Comprehensive Cancer Network (NCCN) guidelines [7-11].

Most of the existing validation and correlation studies for Decipher GC have been conducted using radical prostatectomy specimens, where tumor tissue is more abundant and histopathologic parameters can be assessed comprehensively across the entire gland [7-9]. In contrast, diagnostic biopsy specimens are limited by tissue sampling and heterogeneity. Consequently, the relationship between routinely assessed histopathologic features in biopsy specimens and Decipher GC risk classification remains incompletely understood. Clarifying this relationship in the biopsy setting is clinically important, as it may determine whether readily available pathologic data can inform expectations about genomic risk before definitive therapy, and whether Decipher testing offers independent prognostic value beyond what conventional biopsy parameters already capture.

In this study, we hypothesized that histopathologic markers of tumor burden and aggressiveness identified on diagnostic prostate biopsies would be associated with Decipher genomic classifier groups, reflecting the extent to which routinely available pathologic data correspond with underlying tumor genomic risk in a real-world clinical setting.

## Materials and methods

Prostate biopsy specimens diagnosed as prostatic adenocarcinoma between 2024 and 2025 were retrospectively retrieved from the pathology archives. All biopsies consisted of ultrasound-guided systematic core biopsies with a minimum of six cores. Cases were included if Decipher genomic classifier (GC) testing had been performed.

For each case, hematoxylin and eosin (H&E)-stained slides were reviewed by one genitourinary pathologist and a pathology resident in a blinded manner with respect to the Decipher risk group status. Clinicopathologic data was also collected and included patient age, serum PSA level at diagnosis, the Decipher risk group, grade group (GG), positive core ratio (number of positive cores/total number of cores, per case), maximum tumor length ratio (maximum tumor length/specific core length, per case), presence of cribriform architecture, intraductal carcinoma component, necrosis, neuroendocrine differentiation, extraprostatic extension, metastatic status and sites, and patient vital status. Statistical comparisons were performed across Decipher risk groups.

Statistical analyses were conducted using IBM SPSS Statistics version 28.0.1.0 (Armonk, NY, USA). Continuous variables were compared across the three Decipher risk groups using one-way analysis of variance (ANOVA), with post hoc pairwise comparisons performed using Tukey’s honestly significant difference (HSD) test. PSA levels were compared using the Kruskal-Wallis test, as they are non-normally distributed, with post hoc pairwise comparisons performed using Dunn’s analysis. Categorical variables were analyzed using the chi-square test or Fisher’s exact test, as appropriate, depending on expected cell frequencies. Statistical significance was set at p<0.05 for all analyses.

This study was approved by the Institutional Review Board and Ethics Committee of Albert Einstein College of Medicine, Montefiore Medical Center (approval number: 136724).

## Results

The study cohort comprised 73 prostate adenocarcinoma biopsy cases with available Decipher genomic testing. Based on Decipher results, cases were stratified into three risk categories: low risk (LR; n=28), intermediate risk (IR; n=18), and high risk (HR; n=27).

Although mean age and diagnostic PSA levels showed a gradual increase across the LR, IR, and HR groups, these trends did not reach overall statistical significance (Table 1). However, pairwise analysis demonstrated that the mean age was significantly higher in the HR group compared with the LR group (t=2.17, p=0.03). Given the non-significant omnibus result, this finding should be interpreted as exploratory rather than confirmatory. No significant differences in PSA levels were observed between any risk groups.

**Table 1:** Demographic and clinical characteristics of the cases p-values were calculated using one-way ANOVA (F statistic) and the Kruskal-Wallis test (H statistic). ANOVA: analysis of variance, SD: standard deviation, PSA: prostate-specific antigen, NS: not significant

Variable	Low risk (n=28)	Intermediate risk (n=18)	High risk (n=27)	p-value (test statistic)
Age, mean±SD (years)	63.9±9.4	67.0±7.7	68.5±6.4	0.06 (F=2.35)
PSA, mean±SD (ng/mL)	9.4±5.7	10.1±6.4	10.4±6.4	0.6 (H=0.86)
Metastasis at the time of the study, number (%)	0 (0%)	0 (0%)	1 (3.7%)	NS
Vital status, number (%)	28 alive (100%)	18 alive (100%)	27 alive (100%)	NS

Grade group (GG) distribution differed significantly across the Decipher risk groups (χ²=16.6, p = 0.03). In the LR group, there were 6 GG1, 20 GG2, 1 GG3, and 1 GG5 cases; in the IR group, there were 1 GG1, 13 GG2, 3 GG3, and 1 GG4 cases; and in the HR group, there were 1 GG1, 13 GG2, 9 GG3, 3 GG4, and 1 GG5 cases. The majority of cases across all groups were GG2. Pairwise comparisons demonstrated a significant difference between the HR and LR groups (χ²=12.8, p=0.006), primarily driven by a higher proportion of GG3 tumors in the HR group and a higher proportion of GG1 tumors in the LR group. A near-significant difference was noted between the IR and LR groups (p=0.05), primarily driven by the higher prevalence of GG3 in the IR group. No significant difference was observed between the HR and IR groups (Table 2 and Table 3).

**Table 2:** Histopathologic characteristics of the cases P-values for continuous variables (positive core ratio and maximum tumor length ratio) were calculated using one-way ANOVA (F statistic). P-values for categorical variables were calculated using Pearson’s chi-square test (χ²) when all expected cell counts were ≥5 (cribriform pattern and perineural invasion) or Fisher’s exact test (Freeman-Halton extension for tables larger than 2×2) when any expected cell count was <5 (grade group, extraprostatic extension, and intraductal carcinoma). Necrosis and neuroendocrine differentiation were not statistically tested as no events were observed in any risk group. Percentages are calculated within each risk group and may not sum exactly to 100% because of rounding. ANOVA: analysis of variance, SD: standard deviation, NS: not significant

Variable	Low risk (n=28)	Intermediate risk (n=18)	High risk (n=27)	p-value (test statistic)
Grade group 1, number (%)	6 (21.4%)	1 (5.6%)	1 (3.7%)	0.03 (Fisher’s exact, Freeman-Halton)
Grade group 2, number (%)	20 (71.4%)	13 (72.2%)	13 (48.1%)	
Grade group 3, number (%)	1 (3.6%)	3 (16.7%)	9 (33.3%)	
Grade group 4, number (%)	0 (0%)	1 (5.6%)	3 (11.1%)	
Grade group 5, number (%)	1 (3.6%)	0 (0%)	1 (3.7%)	
Positive core ratio, mean±SD	0.44±0.19	0.54±0.29	0.64±0.26	0.01 (F=4.29)
Maximum tumor length ratio, mean ± SD	0.47±0.28	0.51±0.3	0.67±0.27	0.01 (F=4.72)
Extraprostatic extension, number (%)	0 (0%)	0 (0%)	3 (11.1%)	0.07 (Fisher’s exact)
Intraductal carcinoma, number (%)	0 (0%)	0 (0%)	3 (11.1%)	0.07 (Fisher’s exact)
Cribriform pattern, number (%)	7 (25.0%)	7 (38.9%)	12 (44.4%)	0.3 (χ²=2.37)
Perineural invasion, number (%)	4 (14.3%)	5 (27.8%)	7 (25.9%)	0.4 (χ²=1.56)
Necrosis, number (%)	0 (0%)	0 (0%)	0 (0%)	NS
Neuroendocrine differentiation, number (%)	0 (0%)	0 (0%)	0 (0%)	NS

**Table 3:** Pairwise statistical comparisons across Decipher risk groups Post hoc pairwise comparisons were conducted using Tukey’s HSD test (t statistic) or Dunn’s test with Bonferroni correction (Z statistic). HSD: honestly significant difference, LR: low risk, IR: intermediate risk, HR: high risk, PSA: prostate-specific antigen

Variable	LR versus IR	LR versus HR	IR versus HR	Overall p-value (test statistic)
Age (years)	p=0.28 (t=1.08)	p=0.03 (t=2.17)	p=0.59 (t=0.54)	p=0.06 (F=2.35)
PSA (ng/mL)†	Adj. p=1.000 (Z=-0.359)	Adj. p=0.052 (Z=-2.460)	Adj. p=0.204 (Z=-1.825)	p=0.6 (H=0.86)
Grade group distribution	p=0.05 (Fisher’s exact)	p=0.006 (Fisher’s exact)	p=0.14 (Fisher’s exact)	p=0.03 (Fisher’s exact)
Positive core ratio	p=0.21 (t=1.27)	p=0.003 (t=3.10)	p=0.18 (t=1.36)	p=0.01 (F=4.29)
Maximum tumor length ratio	p=0.71 (t=0.37)	p=0.01 (t=2.64)	p=0.09 (t=1.73)	p=0.01 (F=4.72)

The Decipher risk groups also demonstrated significant differences in mean positive core ratio and maximum tumor length ratio (both F≈4.2-4.8, p=0.01). The mean positive core ratios were 0.44, 0.54, and 0.64 for the LR, IR, and HR groups, respectively, with the HR group showing a significantly higher proportion of positive cores compared with the LR group (t=3.10, p=0.003). Similarly, the mean maximum tumor length ratios were 0.47, 0.51, and 0.67 for the LR, IR, and HR groups, respectively. The HR group demonstrated significantly greater tumor length compared with the LR group (t=2.64, p=0.01) (Table 2 and Table 3).

Aggressive histopathologic features, including the presence of cribriform pattern, extraprostatic extension, perineural invasion, intraductal component, necrosis, and neuroendocrine differentiation, showed no statistically significant differences across the Decipher risk groups.

**Figure 1:**
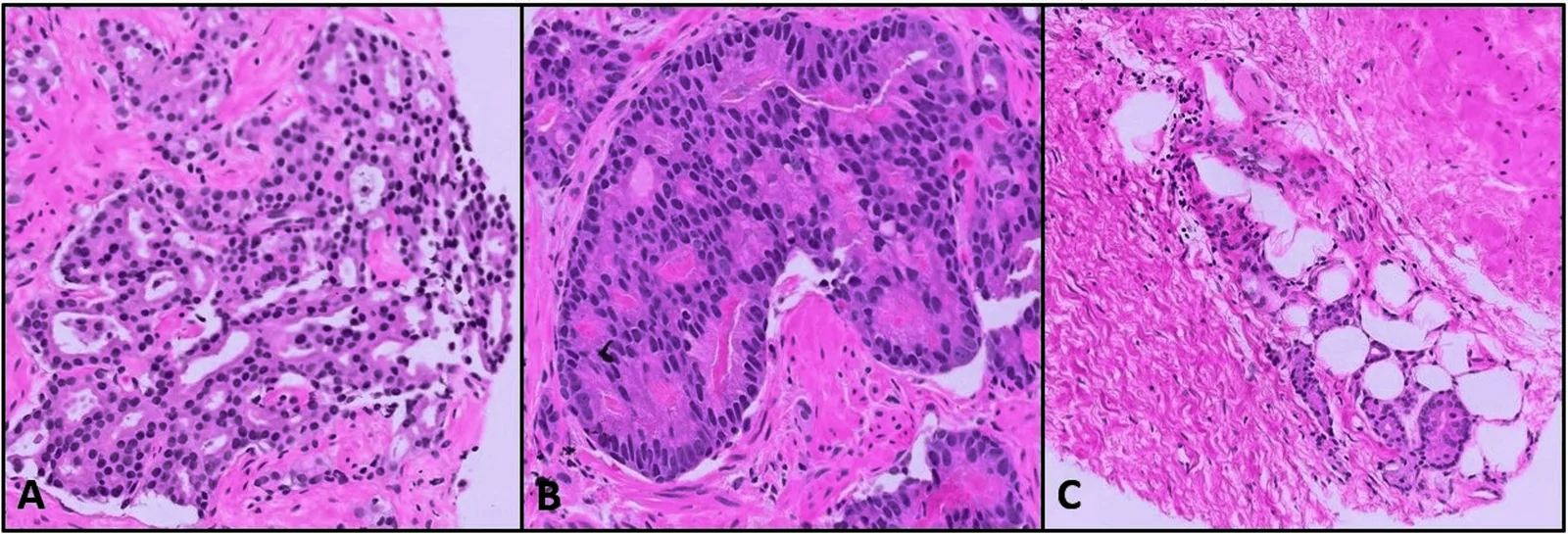
Representative histopathologic features observed in high-risk Decipher group cases (A) Cribriform pattern. (B) Intraductal carcinoma. (C) Extraprostatic extension.

Figure 1 presents histopathologic features observed in high-risk Decipher group cases.

Regarding clinical outcomes, only a single instance of metastasis was documented, which occurred within the high-risk (HR) group. No cancer-related mortality or other patient deaths were recorded during the study period.

## Discussion

Our findings demonstrate that Decipher risk groups are significantly associated with critical histopathologic parameters in diagnostic prostate biopsies, including grade group, positive core ratio, and maximum tumor length ratio.

Historically, prostate cancer management has relied upon clinical risk stratification to guide treatment and surveillance decisions. The National Comprehensive Cancer Network (NCCN) guidelines represent the most widely utilized framework for this purpose, categorizing patients into low, favorable intermediate, unfavorable intermediate, high, and very-high risk groups. These classifications are based on established clinicopathologic features, such as clinical T-stage, grade group, serum PSA levels, and a positive core ratio cutoff of 50% [11].

In recent years, the oncology landscape has shifted toward personalized medicine through the development of multi-gene genomic classifiers. In the context of prostate cancer, these assays have shown substantial promise in refining prognosis and optimizing management. While these tools were initially viewed as adjuncts, they are increasingly being integrated into standard clinical practice guidelines [12]. Notably, within the current NCCN framework, the Decipher classifier (GenomeDx Biosciences, Vancouver, BC, Canada) is the only genomic classifier recommended under the advanced diagnostic tools for risk stratification and biomarker assessment [11]. Our study reinforces this integration by showing that genomic risk groups align with histomorphological aggressiveness of tumors.

The Decipher classifier is a tissue-based platform that evaluates 22 differential genes involved in critical biological pathways, including cellular proliferation, differentiation, immune modulation, and androgen-receptor signaling, derived from whole-transcriptome analysis. It assigns each case a score from 0 to 1 and classifies them into low-risk (LR) (<0.45), intermediate-risk (IR) (≥0.45-<0.6), or high-risk (HR) (≥ 0.6) categories. The assay has been extensively validated as both a prognostic and predictive biomarker across multiple clinical endpoints, such as adverse pathology at radical prostatectomy, biochemical recurrence, metastasis, and prostate cancer-specific mortality following treatment [13,14]. Research has established that Decipher independently predicts oncological outcomes; for instance, patients with low genomic classifier (GC) scores exhibit significantly lower 10-year metastasis rates compared to those with high GC scores (4% versus 16%) following radiotherapy alone. Such data suggest a potential for treatment de-escalation, particularly by omitting short-term androgen deprivation therapy (ADT) in select NCCN intermediate-risk patients [11,15-17]. However, while its utility in surgical specimens is well-documented, there remains a comparative paucity of data regarding its application and association with histopathology in the diagnostic or follow-up biopsy settings [13].

Within our institution’s genitourinary pathology practice, we have observed a marked increase in the clinical utilization of the Decipher genomic assay in the biopsy setting. The primary clinical driver for this trend is the need to distinguish between biologically indolent disease and high mortality phenotypes at an early diagnostic stage, a distinction that conventional prognostic markers, including Gleason scoring, may not sufficiently provide [18].

While our study established that grade groups (GG) correlate significantly with Decipher risk groups, this association was primarily driven by the differences between HR and LR cohorts. Notably, GG alone failed to predict a categorical difference between the IR and HR groups. In alignment with the validation cohort by Kim et al., the majority of biopsies in our study featured lower-grade groups and were subsequently classified into the LR genomic group [19]. However, we identified exceptional cases, such as a GG1 case in the HR group and a GG5 case in the LR group; based on findings by Shee et al., such discordant cases are expected to exhibit clinical behavior dictated more by their genomic profile than their histopathology over the course of clinical care [20].

Our data also demonstrate that positive core ratios correlate with Decipher risk groups. Specifically, the HR group exhibited the highest positive core ratios, a finding that aligns with Vince et al., who reported an increase in percent positive cores within genomic HR groups [16]. Although percent positive cores is a standard variable in traditional NCCN risk stratification, our findings emphasize that these histopathologic differences are most pronounced when comparing the polar ends of the spectrum (HR versus LR) rather than correlating as a seamless three-tier predictive system together with the genomic classifier.

Additionally, our analysis revealed a significant association between the maximum tumor length ratio and Decipher risk groups, which is a relationship that, to our knowledge, has not been previously detailed in the literature. Much like our other findings, this association was predominantly driven by the divergence between the HR and LR groups.

Finally, although not yet fully incorporated into established clinical predictive models, two aggressive histologic features, extraprostatic extension and intraductal carcinoma, were observed exclusively in the high-risk (HR) group. Additionally, other adverse features, including cribriform architecture and perineural invasion, were more prevalent in the HR cohort. The lack of statistical significance for these differences is likely attributable to the limited sample size. As previously established, the presence of these histomorphologic features is strongly associated with adverse clinical outcomes and treatment resistance [21-24]. Our findings suggest that their presence on biopsy may be a potential morphological surrogate for high genomic risk, warranting evaluation in larger cohorts.

Overall, our study underscores the clinical relevance of histopathologic findings and their significant association with genomic classifier risk groups. Given the well-established biological significance of these histopathologic features, these findings support the biological plausibility of the Decipher genomic classifier in the biopsy setting. At the same time, they also reaffirm the enduring validity and critical importance of histomorphology in prostate cancer management; traditional features remain highly predictive of biological behavior, particularly when evaluated within a two-tier genomic risk framework.

Limitations

Despite the clinical relevance of our findings, this study is not without limitations. First, the cohort included only patients who underwent Decipher genomic classifier (GC) testing, introducing potential selection bias, as Decipher is typically ordered for selected patients in whom additional risk stratification is clinically indicated. Consequently, this cohort may not be fully representative of all patients with biopsy-proven prostatic adenocarcinoma, potentially limiting the generalizability of our findings. Second, the retrospective design and relatively modest cohort size (N=73) may have limited the statistical power to detect associations involving less common histopathologic features, such as neuroendocrine differentiation or tumor necrosis. Third, because the study focused on recently diagnosed cases, the follow-up duration was insufficient, precluding evaluation of clinically meaningful long-term outcomes such as biochemical recurrence, metastasis-free survival, prostate cancer-specific survival, and overall survival. Finally, as this was a single-institution study, further multicenter prospective investigations with larger datasets are warranted to validate these associations and facilitate the seamless integration of the Decipher genomic classifier into routine pathologic practice.

## Conclusions

This study indicates that the established histopathologic markers of tumor burden and aggressiveness in diagnostic prostate biopsy specimens significantly correlate with Decipher genomic classifier risk groups. Specifically, higher genomic risk scores were associated with advanced grade groups, increased positive core ratios, and notably longer maximum tumor length ratios. While traditional clinicopathologic variables remain foundational to risk stratification, our findings highlight the ability of genomic profiling to identify high-risk biological phenotypes that may not be fully captured by morphology alone, as evidenced by the presence of high-risk genomic scores in lower-grade specimens.

Furthermore, the exclusive presence of extraprostatic extension and intraductal carcinoma within the high-risk group suggests these features may serve as valuable morphological indicators of a high-risk genomic profile. In conclusion, the integration of Decipher testing into routine clinical practice provides a more nuanced understanding of tumor biology at the time of biopsy, potentially allowing for more personalized treatment pathways and better identification of patients who may benefit from intensified therapy versus those suitable for active surveillance.
